# Brain Functional Network Architecture Reorganization and Alterations of Positive and Negative Affect, Experiencing Pleasure and Daytime Sleepiness in Cataract Patients after Intraocular Lenses Implantation

**DOI:** 10.3390/brainsci11101275

**Published:** 2021-09-26

**Authors:** Anna Maria Sobczak, Bartosz Bohaterewicz, Magdalena Fafrowicz, Aleksandra Zyrkowska, Natalia Golonka, Aleksandra Domagalik, Ewa Beldzik, Halszka Oginska, Marek Rekas, Dominik Bronicki, Bozena Romanowska-Dixon, Joanna Bolsega-Pacud, Waldemar Karwowski, Farzad Farahani, Tadeusz Marek

**Affiliations:** 1 Department of Cognitive Neuroscience and Neuroergonomics, Institute of Applied Psychology, Jagiellonian University, 30-348 Kraków, Poland; vonfrovitz@gmail.com (M.F.); zyrkowska.aleksandra@gmail.com (A.Z.); n.a.golonka@gmail.com (N.G.); ewa.beldzik@uj.edu.pl (E.B.); halszka.oginska@gmail.com (H.O.); tademarek@gmail.com (T.M.); 2 Malopolska Centre of Biotechnology, Jagiellonian University, 30-387 Kraków, Poland; aleksandra.domagalik@uj.edu.pl; 3 Department of Psychology of Individual Differences, Psychological Diagnosis, and Psychometrics, Institute of Psychology, University of Social Sciences and Humanities, 03-815 Warsaw, Poland; 4 Ophthalmology Department, Military Institute of Medicine, 04-349 Warsaw, Poland; rekaspl@gmail.com (M.R.); dominikbronicki@wp.pl (D.B.); 5 Department of Ophthalmology and Ocular Oncology, Medical College, Jagiellonian University, 31-008 Kraków, Poland; bozena.romanowska-dixon@uj.edu.pl (B.R.-D.); joanna.bolsega@gmail.com (J.B.-P.); 6 Computational Neuroergonomics Laboratory, Department of Industrial Engineering & Management Systems, University of Central Florida, Orlando, FL 32816, USA; wkarwowski@gmail.com (W.K.); farzad.vasheghani@knights.ucf.edu (F.F.); 7 Biostatistics Department, John Hopkins University, Baltimore, MD 21218, USA

**Keywords:** neuroimaging, cataract, IOL, fMRI, FC, ALFF, fALFF, affect, pleasure, sleepiness

## Abstract

**Background:** Cataracts are associated with progressive blindness, and despite the decline in prevalence in recent years, it remains a major global health problem. Cataract extraction is reported to influence not only perception, attention and memory but also daytime sleepiness, ability to experience pleasure and positive and negative affect. However, when it comes to the latter, the magnitude and prevalence of this effect still remains uncertain. The current study aims to evaluate the hemodynamic basis of daytime sleepiness, ability to experience pleasure and positive and negative affect in cataract patients after the intraocular lens (IOL) implantation. **Methods:** Thirty-four cataract patients underwent resting-state functional magnetic resonance imaging evaluation before and after cataract extraction and intraocular lens implantation. Both global and local graph metrics were calculated in order to investigate the hemodynamic basis of excessive sleepiness (ESS), experiencing pleasure (SHAPS) as well as positive and negative affect (PANAS) in cataract patients. **Results:** Eigenvector centrality and clustering coefficient alterations associated with cataract extraction are significantly correlated with excessive sleepiness, experiencing pleasure as well as positive and negative affect. **Conclusions:** The current study reveals the hemodynamic basis of sleepiness, pleasure and affect in patients after cataract extraction and intraocular lens implantation. The aforementioned mechanism constitutes a proof for changes in functional network activity associated with postoperative vision improvement.

## 1. Introduction

The state of vision is proved to be strongly associated with perception; attention; executive functions; and learning, memory and motor functioning [1,2,3,4]. Deterioration in vision commonly affects physical, mental and social functioning in patients with cataract disease [5,6,7], whereas cataract extraction and intraocular lens implantation (IOL) have been shown to significantly improve all the aforementioned domains [8]. Cataract disease makes natural lenses yellower and less trasmittent to light, especially in its lower wave range [9,10]. As a result, blue light is filtered out from reaching the eye and its structures, which influences photosensitive ganglion cells (pRGC). It is noteworthy that pRGC is well known for producing melanopsin and taking part in circadian regulation of the body [11]. Following the discovery of the aforementioned mechanism, many experiments involving cataract patients were conducted. Most studies report direct beneficial effects of cataract extraction on excessive sleepiness. Excessive sleepiness is a physiological state that can be defined as compulsion to sleep, taking involuntary naps or experiencing sleep attacks when it is not desired [12]. The above symptoms may accompany different conditions like anemia, hypothyroidism or sleep apnea but could also result from lifestyle, including chronic sleep deprivation, shift work or an irregular sleep pattern [13]. Excessive daytime sleepiness and other sleep-related problems are often observed in the population of older people from all geographical and cultural backgrounds [14]. This phenomenon is believed to be caused by a diverse set of factors consisting of normal aging mechanisms, several conditions prevalent in older populations such as depression or restless leg syndrome, and side effects of pharmacological treatments [15]. However, there is also a growing body of research linking daytime sleepiness and sleep disturbances in the elderly with cataracts [16,17,18,19,20]. Asplund and Lindbald [18] found that the proportion of participants experiencing poor sleep was smaller 9 months after cataract surgery than 1 month after. Similarly, Schenshen et al. [20] found that ESS scores of cataract patients significantly decreased a month after surgery. However, Erichsen and colleagues [17] concluded that daytime sleepiness measured by ESS did not change significantly after surgery. Mixed results were also presented by Schmoll and colleagues [19], who reported improved ESS scores after the surgery of the first eye but not after the second one. There is evidence on the short-term beneficial effect of cataract surgery on daytime sleepiness, yet the magnitude and prevalence of this effect still remains uncertain. An fMRI study conducted on patients with early Parkinson’s disease revealed that excessive daytime sleepiness is associated with increased activation in default mode network [21], while the study on healthy participants showed it is related to altered thalamocortical activity [22]. However, to our best knowledge, no fMRI study investigating daytime sleepiness has yet been made on cataract patients after IOL implantation.

Cataract surgery is also reported to influence the affective state [23]. Research on affect differences in lifespan does not come with clear conclusions. While some publications report an increase in positive affect in the elderly [24,25], most of them suggest either a decrease [26,27,28,29,30] or lack of age-related difference [30]. Similarly for negative affect, various studies showed a decline [27,30,31,32], increase [26,29] or no significant difference [28]. In some cases, the initially demonstrated dependence of age on affect vanished after controlling other variables [33]. Affective experiences seem to be multidimensional; multidirectional [34]; and under the influence of many factors, including medical ones. Most research supports the thesis of the positive impact of cataract surgery on mood [35,36,37], which is usually associated with improved visual acuity [38]. Previous studies on affect suggest that there is an increase of positive affect immediately after surgery and at the six-week follow-up as well as a postoperative decrease of negative affect with a small but significant increment from discharge to six weeks post-surgery [39]. However, some papers postulate that there is no correlation between cataract surgery and mood [40]. Once again, the relationship seems to be more complex as studies report that in patients with only cataracts, mood significantly improves after surgery, while in patients with other comorbidities it deteriorates post-operation [41]. Neuroimaging studies revealed a mainly positive impact of cataract surgery on a general functioning [42,43]. A recent study [42] showed increased grey matter volume of the visual, somatosensory and cognitive brain areas. The above results may derive from an improvement in vision-related quality of life, cognitive decline or depressive state, which are reported to be strongly related with each other [42].

While the attention, time reaction and memory have already been examined in numerous fMRI studies focusing on cataract disease, mood variables such as daily sleepiness, pleasure experience and affectivity after intraocular lens implantation have not been thoroughly explored yet. The current study aims to evaluate the hemodynamic basis of daytime sleepiness, the ability to experience pleasure, and positive and negative affect in cataract patients after IOL implantation.

## 2. Materials and Methods

### 2.1. Participants

Thirty-eight cataract patients qualified for the study; however, four of them had to drop out after the first session due to health issues. Hence, a total of thirty-four participants were subject to fMRI examination before and after cataract extraction and the implantation of an intraocular lens. The mean age of participants was M = 62.3 years old, SD = 9.1, consisting 22 women and 12 men. The participants were subject to fMRI examination two weeks before the surgery and 6–12 months after (M = 9.22; SD = 2.66). The patients were recruited by qualified ophthalmologists after being diagnosed with the cataract in the Polish national healthcare system. The inclusion criteria were being diagnosed with a cataract as well as qualification for the surgery. The exclusion criteria were neurological and psychiatric disorders, lesions, and contraindications for magnetic resonance imaging. The study was approved by the ethics commissions at the Polish Military Institute of Aviation Medicine and Institute of Applied Psychology at the Jagiellonian University, Cracow, Poland (21 February 2017). It was conducted in accordance with ethical standards described in the Declaration of Helsinki. All participants were informed about the procedure and provided their written consent.

### 2.2. Intraocular Lens Implantation

Before IOL implantation, ophthalmologists measured the yellowing of their lenses with the use of fluorophotometry (Ocumetrics Fluorotron Master, Mountain View, CA, USA) or with the Lens Opacities Classification System III (LOCS III). The IOL type was chosen by ophthalmologists. Patients had two types of IOL implanted: with blue light filter transmitting 68% of light around 475 nm (Alcon AcrySof^®^, Geneva, Switzerland, IQ model SN60WF) or crystal lenses transmitting 95% of blue light (HOYA Ltd., Singapore, model iSert 250, Abbott Medical Optics Inc. model Symphony, Alcon AcrySof^®^, Geneva, Switzerland, IQ model AU00T0, Akreos^®^, Bridgewater, NJ, USA, model Adapt AO).

### 2.3. Psychological Questionnaires

#### 2.3.1. The Epworth Sleepiness Scale (ESS)

ESS allows one to measure daytime sleepiness while evaluating the possibility of falling asleep during various daily activities [44]. The questionnaire has a high level of internal consistency with Cronbach’s alpha α = 0.88 [45].

#### 2.3.2. Snaith-Hamilton Pleasure Scale (SHAPS)

SHAPS enables evaluation of the ability to experience pleasure (hedonic tone) as well as its absence (anhedonia) [46]. According to Franken et al. [47], it is highly reliable in terms of internal consistency and test–retest stability.

#### 2.3.3. Positive and Negative Affect Schedule (PANAS)

PANAS is a self-reported 20-item questionnaire, consisting of two 10-item subscales measuring positive and negative affect. The scales were reported to be highly internally consistent, uncorrelated and stable in time [48].

### 2.4. MRI Data Acquisition

MRI data were acquired using 3T Siemens Skyra MR System (Siemens Medical Solutions, Erlangen, Germany). Structural images were obtained with the use of sagittal 3D T1-weighted MPRAGE sequence. Ten minute functional resting state (rs-fMRI) EPI images were acquired using gradient-echo single-shot echo planar imaging sequence with the following parameters: TR = 2000 ms; TE = 27 ms; slice thickness = 3 mm; and voxel size = 3 mm^3^, with no gap using 20-channel coil. Total of 37 interleaved transverse slices and 300 volumes were acquired. During the acquisition, participants were instructed to keep their eyes open and to not think about anything in particular.

### 2.5. Imaging Data Preprocessing

The rs-fMRI data processing was performed using Data Processing and Analysis for Brain Imaging (DPABI) V4.3 [49] and SPM 12 (Wellcome Trust Centre for Neuroimaging, UCL, London, UK), which were working under MATLAB version R2018a (The MathWorks, Inc., Natick, MA, USA). First 10 time points were deleted due to signal equilibration, and then slice timing was conducted. Next, realignment with assessment of the voxel specific head motion was conducted. None of the participants displayed movements above 3 mm or 3° in one or more of the orthogonal directions, and therefore all patients qualified for further analysis. Then, using standard EPI template, functional images were linearly normalized in DARTEL to Montreal Neurological Institute (MNI) space and spatially resampled to 3 × 3 × 3 mm voxel size. The 24-motion parameters were derived from the realignment step, white matter, and cerebrospinal fluid signals, and five principal components were removed using principal components analysis integrated in a component-based noise correction method [50]. The global signal was included because of its potential to provide additional valuable information [51]. The signal was then band-pass filtered (0.01–0.08 Hz) in order to reduce high-frequency noise and low-frequency drift, such as the respiratory and cardiac rhythms. Lastly, the functional data was spatially smoothed with 4 mm Full Width at Half Maximum (FWHM) kernel.

### 2.6. Parcellation

Following preprocessing, data was parcellated with the use of automated anatomical labeling (AAL) atlas, which separates the brain into 116 regions [52]. In order to investigate between-session differences among default-mode network, salience network, basal ganglia network, higher visual network, primary visual network and visuospatial network, the authors used templates from FIND lab (http://findlab.stanford.edu/functional_ROIs.html) Accessed on 15 November 2020.

### 2.7. Graphs Metrics

For the purpose of examining the topological properties of functional brain network for each participant at both global and local levels, Graphvar 2.02b [53] and MATLAB version R2018a (The MathWorks, Inc., Natick, MA, USA) were used. In the present study, both global and local measures were calculated. Global included mean clustering coefficient and assortativity. Moreover, local common properties such as clustering coefficient and eigenvector centrality were also calculated (the measures are discussed in detail in https://sites.google.com/site/bctnet/measures/list). Accessed on 15 November 2020. Data used for graph measures were not smoothed during preprocessing steps. For each subject, 116 regions of interest (ROIs) were defined according to the AAL atlas [52]. In order to obtain a 116 × 116 undirected binary correlation matrix, mean-time course for each region was extracted, and then the Pearson coefficients between each pair of ROIs were calculated. In order to exclude the spurious links in interregional connectivity matrices [54], we adopted a thresholding procedure based on the strongest connections, removing the weaker ones [55]. This procedure enables us to compare network topology within as well as between participants [56]. Network edges were defined using a sparsity thresholding procedure ranging from 0.1 to 0.5 in steps of 0.05.

### 2.8. Statistical Analysis

Paired *t*-test was used while comparing graph indexes before and after cataract extraction. The results were calculated with 5000 iterations and corrected with the Benjamini and Hochberg [57] false discovery rate correction at *p* < 0.05. Paired *t*-test with 5000 iterations, as well as non-parametric FDR-corrected *p*-value < 0.05, was conducted using Graphvar 2.02 [53] and MATLAB version R2018a (The MathWorks, Inc., Natick, MA, USA).

Pearson correlation was calculated in order to investigate the association between significant between-session differences in global and local functional network architecture and variables associated with excessive sleepiness, experiencing pleasure and affect. The psychological variables that were correlated with fMRI data were all the differences between the first and the second session. The differences were calculated in such a manner that the positive value would always indicate improvement (for example, lower level of daytime sleepiness, negative affect, and anhedonia, as well as higher level of positive affect and pleasure). By analogy, the negative value in the difference between pre- and postoperative state meant worsening in terms of sleepiness, mood and experiencing pleasure. Only significant differences in graph indexes were correlated with aforementioned psychological features. Mean differences as well as standard deviations of investigated variables, which were later correlated with significant between-session graph differences, are presented in Table 1.

## 3. Results

Pearson correlation revealed significant positive association between eigenvector centrality in left cerebellum VIII, bilateral superior parietal gyrus, right supramarginal gyrus and differences in ESS values, as well as negative correlation between eigenvector centrality in left cerebellum VIII and left cerebellum VIIb, and differences in PANAS positive affect and SHAPS pleasure values.

Moreover, Pearson correlation showed positive association between clustering coefficient in right superior parietal gyrus and difference in SHAPS anhedonia values. Negative correlation was observed between difference in PANAS positive affect and clustering coefficient in supplementary motor area as well as Vermis VIII.

Table 2 depicts ROIs with significant between-session differences that correlate with EES, PANAS and SHAPS values. Table 3 and Table 4 show correlation values between significant between-session differences in graph indexes and excessive sleepiness, experiencing pleasure, and positive and negative affect.

**Figure 1:**
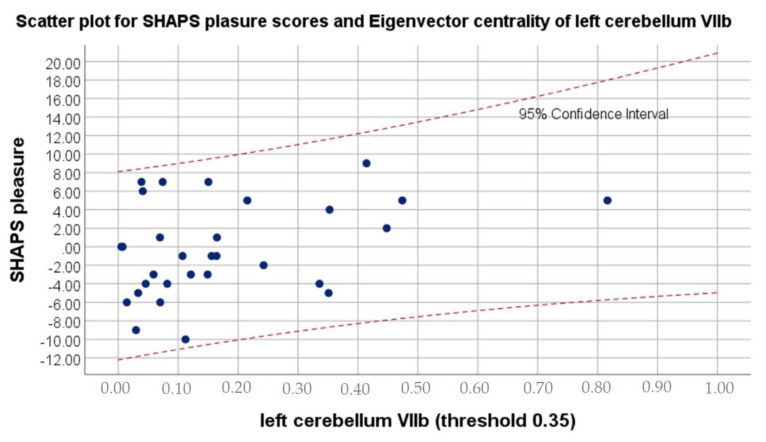
Scatter plot for SHAPS pleasure scores and Eigenvector centrality values on 0.35 threshold of left cerebellum VIIb.

**Figure 2:**
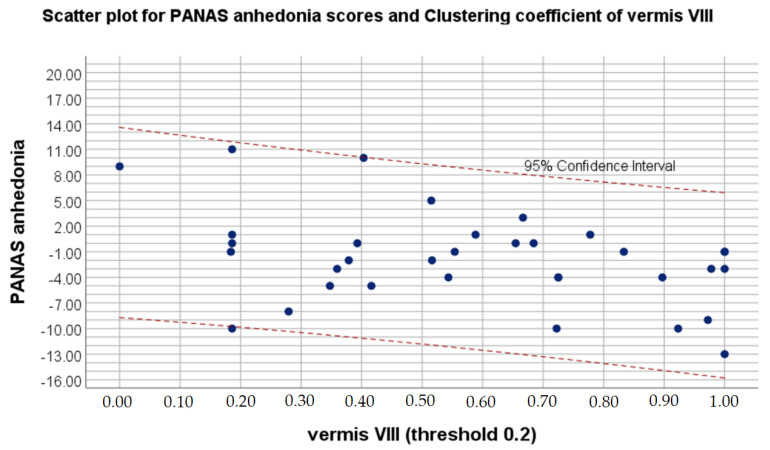
Scatter plot for PANAS anhedonia scores and clustering coefficient on 0.2 threshold of vermis VIII.

**Figure 3:**
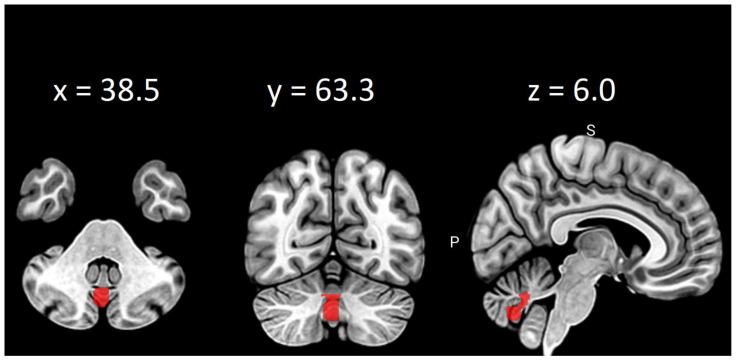
Clustering coefficient differences in vermis VIII on thresholds: 0.2, 0.25, 0.30 and 0.35.

Figure 1 shows the strongest correlation of psychological features with significant between-session alterations in eigenvector centrality, while Figure 2 visualizes strongest relation of psychological variable with significant differences in clustering coefficient. Figure 3 presents localization of alterations in Vermis VIII, which showed different clustering coefficient values for four thresholds in a row.

## 4. Discussion

Current study showed cataract-surgery-induced changes in positive affect, ability to experience pleasure and daytime sleepiness. Moreover, hemodynamic basis of above alterations was revealed. The results of graph analysis indicated between-session differences in eigenvector centrality and clustering coefficient. Clustering coefficient measures the degree to which regions of the brain tend to cluster together and form a functional network, but at the same time it represents its local integration. A network with higher clustering coefficient is considered to be a common characteristic of the healthy brain as it allows one to differentiate between people with and without neurodegenerative diseases [58]. In addition, the clustering coefficient is believed to decrease with age, due to the lower effectiveness of the brain, associated with a gradual loss of synapses [59]. Eigenvector centrality, on the other hand, is a self-referential measure of centrality. Higher value is equivalent to the greater importance of the region for the whole network as well as being connected with other influential regions. Moreover, eigenvector centrality is reported to be positively correlated with the firing rate of the neuron [60]. Meanwhile, recent studies suggest increased relative firing rate to be distinctive for progressive loss of synapses. It constitutes a coping, compensatory mechanism that allows one to avert the disruption of the functional network [61,62]

In the present study, smaller postoperative eigenvector centrality in the left cerebellum VIII was associated with a higher level of positive affect. Cerebellum VIII is known for contributing to somatosensory and motor processing [63]. Cataract extraction should positively influence both sensorimotor reactions and psychomotor functions, regardless of the type of implant lens [64]. Therefore, postoperative decrease of compensatory mechanisms is congruent with previous reports on eigenvector centrality connotations. Moreover, smaller clustering coefficient values in supplementary motor area after cataract extraction and IOL implantation were also related to higher level of positive affect. While clustering coefficient is associated with segregation and integration of the network, decrease in above value can suggest disorganization of the functional neural network as well as interfering supplementary motor area activity. Noteworthily, functional alterations of above structure have been disclosed in patients complaining of excessive fatigue [65,66,67]. Previous study by Zénon, Sidibé and Olivier [68] revealed that disrupting the aforementioned structure makes physical effort appear less exhausting. In addition, noninvasive neurodisruption of supplementary motor area is reported as a potential therapeutic procedure for treating pathological fatigue associated with various disorders, such as multiple sclerosis, traumatic brain injury or cataract disease [68]. At the same time, the current study presented increased postoperative clustering coefficient in vermis VIII to be related to a declined level of positive affect. Even though it may appear surprising that higher integration of the network is associated with worsened mood, larger volume of vermis VIII in elderly people was found to be positively correlated with the high scores on the lack of positive affect subscale [69]. Above findings support the relationship between depressive symptoms and posterior cerebellum.

The current study also revealed smaller eigenvector centrality in the left cerebellum VIIb to be associated with greater capability to experience pleasure. Cerebellum VIIb is reported to be responsible for executive functions in diverse domains such as emotional processing, language and spatial processing [63]. Better execution usually means increased efficacy and as a result higher overall satisfaction, even in everyday activities. Noteworthy, lobules VII and VIII of cerebellum are particularly known to support sensorimotor, cognitive and emotional functions [70]. Therefore, decreased postoperative need for compensatory mechanisms in cerebellum VIIb might be associated with improved overall functioning as well as experiencing pleasure. The above results are congruent with the following ones, referring to the relationship between higher clustering coefficient in the right superior parietal gyrus and lower level of anhedonia after the cataract extraction. Lin et al. [43] strongly suggest that alterations in above structure might be associated with visual restoration and functional recovery after cataract extraction, hence it can also be related to decrease in anhedonia.

In addition, decreased postoperative eigenvector centrality in the left cerebellum VIIb, bilateral superior parietal gyrus and right supramarginal gyrus were associated with higher level of daytime sleepiness. Above results contradict previous studies on excessive sleeping after cataract extraction. As mentioned before, most of them point to a short time beneficial effect or no effect at all [17,19]. It appears that the relationship between daytime sleepiness and cataract extraction still remains uncertain. Perhaps it includes other mediators or moderators that should be further investigated.

## 5. Conclusions

In conclusion, the current study revealed the hemodynamic basis of excessive sleepiness, positive affect, pleasure and anhedonia after cataract extraction and IOL implantation. The results indicate surgery’s positive influence on mood and experiencing pleasure; however, postoperative increased daytime sleepiness does not correlate with previous literature; therefore, the above matter still remains uncertain. Further study should focus on extending the sample size and collecting information about daytime sleepiness at many more time points in order to clarify postoperative alterations in redundant sleepiness.

## 6. Limitation

The current study has the restricted sample size, and it is not equivalent in the case of gender. Moreover, only four global and local graph properties were calculated. Future studies should consider addressing these concerns.

## Figures and Tables

**Table 1:** Mean between group differences in the investigated psychological values. Positive value always indicates improvement, whereas negative values indictae deterioration.

Variable	Values	
	M	SD
difference in ESS (session 1–session2)	0.2	3.38
difference in SHAPS pleasure (session 2–session 1)	−0.68	5.06
difference in SHAPS anhedonia (session 1–session 2)	0.48	1.56
difference in PANAS positive affect (session 2–session 1)	−1.85	5.48
difference in PANAS negative affect (session 1–session 2)	−0.56	4.34

**Table 2:** List of brain ROIs with significant between-session differences in clustering coefficient and eigenvector centrality values, which correlate with ESS, SHAPS and PANAS values.

			*p*-Value	
ROI (Names)	AAL Label	Threshold	Clustering Coefficient	Eigenvector Centrality
**Preoperative > Postoperative**				
**Right Supplementary** **Motor Area**	SMA.R	0.4	0.027	
**Left Superior Parietal** **Gyrus**	SPG.L	0.15		0.042
**Right Superior Parietal** **Gyrus**	SPG.R	0.15		0.016
**Right Supramarginal** **Gyrus**	SMG.R	0.35		0.029
**Left Cerebellum VIIb**	CER7b.L	0.35		0.024
**Left Cerebellum VIII**	CER8.L	0.1		0.03
**Postoperative > Preoperative**				
**Right Superior Parietal** **Gyrus**	SPG.R	0.5	0.046	
**Vermis VIII**	VER8	0.2	0.033	
		0.25	0.034	
		0.3	0.023	
		0.35	0.025	

**Table 3:** Significant correlations between significant between-session differences in eigenvector centrality and psychological features.

	Eigenvector Centrality	
**SHAPS pleasure**	**left cerebellum VIIb (threshold 0.35)**	
	**r**	−0.4
	***p***	0.021
**ESS**	**left cerebellum VIIb (threshold 0.1)**	
	**r**	0.36
	***p***	0.036
	**left superior parietal gyrus (threshold 0.15)**	
	**r**	0.34
	***p***	0.048
	**right superior parietal gyrus (threshold 0.15)**	
	**r**	0.39
	***p***	0.023
	**right supramarginal gyrus (threshold 0.35)**	
	**r**	0.37
	***p***	0.033
**PANAS** **positive affect**	**left cerebellum VIII (threshold 0.1)**	
	**r**	−0.36
	***p***	0.036

**Table 4:** Significant correlations between significant between-session differences in clustering coefficient and psychological features.

	Clustering Coefficient	
**SHAPS anhedonia**	**right superior parietal gyrus (threshold 0.5)**	
	**r**	0.34
	***p***	0.047
**PANAS** **positive affect**	**vermis VIII (threshold 0.2)**	
	**r**	−0.4
	***p***	0.02
	**vermis VIII (threshold 0.25)**	
	**r**	−0.35
	***p***	0.044
	**vermis VIII (threshold 0.3)**	
	**r**	−0.37
	***p***	0.029
	**vermis VIII (threshold 0.35)**	
	**r**	−0.4
	***p***	0.028
	**right supplementary motor area (threshold 0.4)**	
	**r**	−0.4
	***p***	0.02

## Data Availability

The data can be shared upon the request.
